# Epidemiological and histological findings implicate matrix Gla protein in diastolic left ventricular dysfunction

**DOI:** 10.1371/journal.pone.0193967

**Published:** 2018-03-12

**Authors:** Fang-Fei Wei, Sander Trenson, Pierre Monney, Wen-Yi Yang, Menno Pruijm, Zhen-Yu Zhang, Yassine Bouatou, Qi-Fang Huang, Belen Ponte, Pierre-Yves Martin, Lutgarde Thijs, Tatiana Kuznetsova, Karel Allegaert, Stefan Janssens, Cees Vermeer, Peter Verhamme, Michel Burnier, Murielle Bochud, Georg Ehret, Jan A. Staessen

**Affiliations:** 1 Studies Coordinating Centre, Research Unit Hypertension and Cardiovascular Epidemiology, Department of Cardiovascular Sciences, University of Leuven, Leuven, Belgium; 2 Division of Cardiology, University Hospitals Leuven, Leuven, Belgium; 3 Department of Cardiology, Centre Hospitalier Universitaire Vaudois, University of Lausanne, Lausanne, Switzerland; 4 Department of Nephrology, University Hospital of Lausanne, Lausanne, Switzerland; 5 Department of Pathology, Academisch Medisch Centrum, Universiteit van Amsterdam, Amsterdam, The Netherlands; 6 Department of Nephrology, University Hospital of Geneva, Geneva, Switzerland; 7 Research Unit Organ Systems, Department of Development and Regeneration, University of Leuven, Leuven, Belgium; 8 Department of Pediatric Surgery, Erasmus Medical Center, Sophia Children’s Hospital, Rotterdam, The Netherlands; 9 R&D Group VitaK, Maastricht University, Maastricht, The Netherlands; 10 Research Unit Molecular and Vascular Biology, Department of Cardiovascular Sciences, University of Leuven, Leuven, Belgium; 11 Division of Chronic Disease, Institute of Social and Preventive Medicine, University Hospital of Lausanne, Lausanne, Switzerland; 12 Department of Cardiology, University Hospital of Geneva, Geneva, Switzerland; 13 Center for Complex Disease Genomics, McKusick-Nathans Institute of Genetic Medicine, John Hopkins University, Baltimore, Maryland, United States of America; Scuola Superiore Sant'Anna, ITALY

## Abstract

**Objectives:**

A novel paradigm of diastolic left ventricular (LV) dysfunction proposes involvement of the cardiac microvasculature. Vitamin K dependent matrix Gla protein (MGP) plays a role in preserving microcirculatory integrity. We hypothesized that LV filling pressure–a measure of diastolic LV dysfunction–increases with higher plasma level of inactive desphospho-uncarboxylated MGP (dp-ucMGP). We also studied the distribution of active and inactive MGP in human myocardium.

**Methods:**

We measured echocardiographic diastolic LV function and plasma dp-ucMGP (ELISA) in 668 Flemish and for replication in 386 Swiss.

**Results:**

Among Flemish and Swiss, E/e’ (6.78 *vs*. 6.73) and dp-ucMGP (3.94 μg/L *vs*. 4.20 μg/L) were similarly distributed. In multivariable-adjusted models, for each doubling of dp-ucMGP, E/e’ increased by 0.26, 0.33 and 0.31 in Flemish, Swiss and both cohorts combined (*P*≤0.026); the odds ratios for having E/e’ ≥ 8.5 were 1.99, 3.29 and 2.36, respectively (*P*≤0.017). Cardiac biopsies from patients with ischemic or dilated cardiomyopathy and healthy hearts (n = 4 for each) were stained with conformation-specific MGP antibodies. In diseased compared with normal hearts, uncarboxylated inactive MGP was more prevalent (*P*≤0.004) in the perivascular matrix and interstitium (204.4 *vs*. 8.6 μm2 per field) and phosphorylated active MGP in and around capillaries and interstitial cells (31.3 *vs*. 6.6 number of positive capillaries and cells per field).

**Conclusions:**

Our study supports a role of activated MGP in maintaining myocardial integrity and diastolic LV performance and can potentially be translated into new strategies for managing diastolic LV dysfunction and preventing its progression to heart failure.

## Introduction

In view of the demographic transition, heart failure (HF) is a major public health problem [1]. Diastolic HF, also referred to as HF with preserved ejection fraction, accounts for 50% of cases [2]. Mortality of diastolic HF is 30% within one year of the first hospital admission [3]. Subclinical diastolic left ventricular (LV) dysfunction has a prevalence of 25% in the general population [4,5], predisposes to further deterioration of LV function [6], and finally to overt HF [2]. A novel paradigm of diastolic HF focuses on proinflammatory signaling originating from the cardiac microvasculature [2]. Matrix Gla protein (MGP) is a small protein (11 kD) synthesized by vascular smooth muscle cells and the endothelium [7]. Activation of MGP requires two posttranslational modifications: vitamin-K dependent γ-glutamate carboxylation and serine phosphorylation [7]. Activated MGP is a potent locally acting inhibitor of calcification in large arteries [8] and protects against macrovascular complications [9,10].

Recent studies support a role of MGP in preserving the integrity of the microcirculation [11] and protecting against calcium deposition [12,13]. Relaxation of the heart requires sequestration of calcium ions in the endoplasmatic reticulum [14]. In view of the novel paradigm implicating the microcirculation in the pathogenesis of diastolic LV dysfunction [2] and the potential system-wide role of MGP [9–13], we hypothesized that echocardiographically assessed diastolic LV function might be inversely associated with inactive circulating desphospho-uncarboxylated MGP (dp-ucMGP). We investigated our hypothesis in a Flemish [13,15] and Swiss [8] population sample. At the time of writing of this manuscript, studies dealing with expression of MGP in the heart were scarce and did not provide any detail where in the myocardium MGP was expressed [16]. After having obtained our initial finding of association between diastolic LV function with circulating dp-ucMGP in Flemish and after having it replicated in Swiss, we undertook histological studies to confirm the presence of MGP in the heart and to determine its exact localization in healthy and diseased hearts, using conformation-specific MGP antibodies.

## Methods

### Population studies

The Flemish Study on Environment, Genes and Health Outcomes (FLEMENGHO) [13,15] and the Swiss KIdney Project On Genes in Hypertension (SKIPOGH) [8] are family-based population studies, which comply with the Helsinki declaration for research in human subjects [17] and received ethical approval. All participants provided informed written consent. FLEMENGHO participants were recruited from a geographically defined area in Northern Belgium. After recruitment from 1985 until 2004 (initial participation rate, 78.0%), participants remained in follow-up. From 2005 until 2014, the re-examination included echocardiography (re-examination rate, 80.3%). Of 964 participants, 696 had dp-ucMGP measured concurrently with echocardiography. The SKIPOGH participants were enrolled from 2009 until 2013 in Berne, Geneva and Lausanne, Switzerland [8]. Of 1129 participants (participation rate, 25.6%), 390 had both echocardiographic images and plasma dp-ucMGP levels available for this analysis. For the current analysis, we excluded 17 Flemish, because of atrial fibrillation (n = 6), paced heart rhythm (n = 2) or poor image quality (n = 9) making assessment of diastolic LV function difficult. In addition, we excluded 11 Flemish and 4 Swiss, because indexes of LV function (n = 10) or MGP levels (n = 5) were more than 3 SDs away from the population mean. Thus, the number of participants statistically analyzed totaled 668 Flemish and 386 Swiss.

In the two cohorts, echocardiographic images were acquired and analyzed off-line according to current guidelines [18,19], following methods described in detail elsewhere [4]. In short, echocardiographic images were obtained with a Vivid7 Pro device (GE Vingmed, Horten, Norway) interfaced with a 2.5- to 3.5-MHz phased-array probe. For off-line analysis, we used EchoPac software, version 4.0.4 (GE Vingmed, Horten, Norway) and averaged measurements over three heart cycles. We determined the peak early (E) and peak late (A) diastolic velocities of the transmitral blood flow from the pulsed Doppler signal and peak early (e’) and peak late (a’) velocities of the mitral annular movement by tissue Doppler imaging (TDI) with velocities averaged over four acquisition sites (septal, lateral, inferior, and posterior). The intra-observer reproducibility coefficient for the single observer in FLEMENGHO was 4.8% for e’ and 4.2% for a’ [6]. In SKIPOGH (two observers), the intra- and inter-observer reproducibility coefficients were 4.4% and 13.8% for e’ and 5.0% and 12.0% for a’, respectively. To stage diastolic LV function, we combined the velocities of the transmitral blood flow and the mitral annular movement [4]. Patients with diastolic LV dysfunction had an abnormally low age-specific transmitral E/A ratio indicative of impaired relaxation, a mildly-to-moderately elevated LV filling pressure (E/e' >8.5) with normal or decreased age-specific E/A ratio. These age-specific criteria in a healthy reference sample drawn from FLEMENGHO [4] were replicated in an independent European population study [5].

Blood pressure was the average of five consecutive auscultatory readings obtained with a standard mercury sphygmomanometer in FLEMENGHO [13,15] and with a non-mercury manual auscultatory sphygmomanometer (A&D UM-101; A&D Company Ltd, Toshima Ku, Tokyo, Japan [20]) in SKIPOGH [8]. Nurses administered questionnaires inquiring into each participant’s medical history, smoking and drinking habits, and intake of medications. Fasting blood samples were analyzed for glucose and total cholesterol, using automated methods in certified laboratories. In both cohorts, dp-ucMGP was measured by VitaK (Maastricht University, The Netherlands) on citrated plasma by pre-commercial ELISA kits [21].

For database management and statistical analysis, we used the SAS system, version 9.4 (SAS Institute Inc., Cary, NC). Significance was a two-tailed α-level of 0.05 or less. Means were compared using the large-sample z-test and proportions by Fisher’s exact test. We normalized the distribution of dp-ucMGP by a logarithmic transformation. We compared cohort-, sex- and age-standardized echocardiographic measurements across halves of the dp-ucMGP distribution. We applied mixed models and logistic regression to model the multivariable-adjusted associations of continuous or categorical LV traits with dp-ucMGP, while accounting for clustering within families and cohort as random effects, as appropriate. Covariables with potential relevance have been described in previous publications [4–6] and included sex, age, body mass index, mean arterial pressure, pulse pressure, heart rate, serum total cholesterol, plasma glucose, left ventricular mass indexed to body surface area, alcohol intake and use of antihypertensive drugs. Analyses including both Flemish and Swiss were additionally adjusted for center.

### Histological studies

Cardiac tissue samples were obtained from patients with end-stage ischemic (ICM, n = 4) or dilated (DCM, n = 4) cardiomyopathy undergoing cardiac transplantation at the University Hospitals Leuven, Belgium. Control tissue samples were obtained from unused donor hearts (HD, n = 4). The Ethics Committee of the University Hospitals Leuven approved the study (approval numbers B322201421186 [S56384] and B322201421045 [S56472]) and it passes ethical screening by the European Research Council Executive Agency (ERCEA). Patients undergoing transplantation provided informed written consent. Donors of healthy hearts discarded for transplantation were unconscious and could not provide consent. However, their next of kin did provide consent for removal of the donor heart. The identity of the donors remained unknown to the transplant team in Leuven. As outlined above, the Ethics Committee of the University Hospitals Leuven approved the use of discarded donor hearts for research.

Tissue samples were paraffin embedded and stained after antigen retrieval (Dako, Glostrup, Denmark) and blocking steps with methanol + 1% H2O2 and goat serum (1:5). Immunofluorescence staining was performed on 6-μm paraffin sections. Images were recorded on a Carl Zeiss LSM700 confocal microscope and analysed with ZEN 2012 software (Jena, Germany). For staining, we used primary mouse anti-human conformation-specific antibodies (VitaK, Maastricht, The Netherlands) specifically directed against uncarboxylated MGP (ucMGP; 1:100), carboxylated MGP (cMGP; 1:250), desphospho-MGP (dpMGP; 1:100) and phosphorylated MGP (pMGP; 1:100). The secondary biotinylated goat anti-mouse antibody Dako, E0433, 1:300) was amplified with Streptavidin and Cy3 (Perkin Elmer, TSA Cy3 system NEL704A001KT; 1:100 and 1:50). An Alexa Fluor® 488 conjugate of wheat germ agglutinin (WGA, W11261, 1:100, Thermo-Fisher Scientific, Waltham, MA) was used as lectin to stain cell membranes and enhance morphological characterization. Endothelium was stained with mouse anti-human CD31 (Dako M0823; 1:50) and smooth muscle cells with a monoclonal mouse anti-human SM actin (M0851, Dako, 1:500). Nuclei were counterstained with TO-PRO-3 (Thermo-fisher, T3605, 1:500). Paraffin sections were stained with hematoxylin and eosin for routine histological analysis and Sirius Red for detection of fibrillar collagen. Staining highlighted uncarboxylated MGP (ucMGP), carboxylated MGP (cMGP), unphosphorylated MGP (dpMGP) and phosphorylated MGP (pMGP). pMGP and cMGP include the secreted and active MGP conformation. Statistical analyses of the histological data were performed with Graphpad Prism 7.0b (La Jolla, CA), using the non-parametric Mann-Whitney test.

## Results

### Population studies

**Fig 1:**
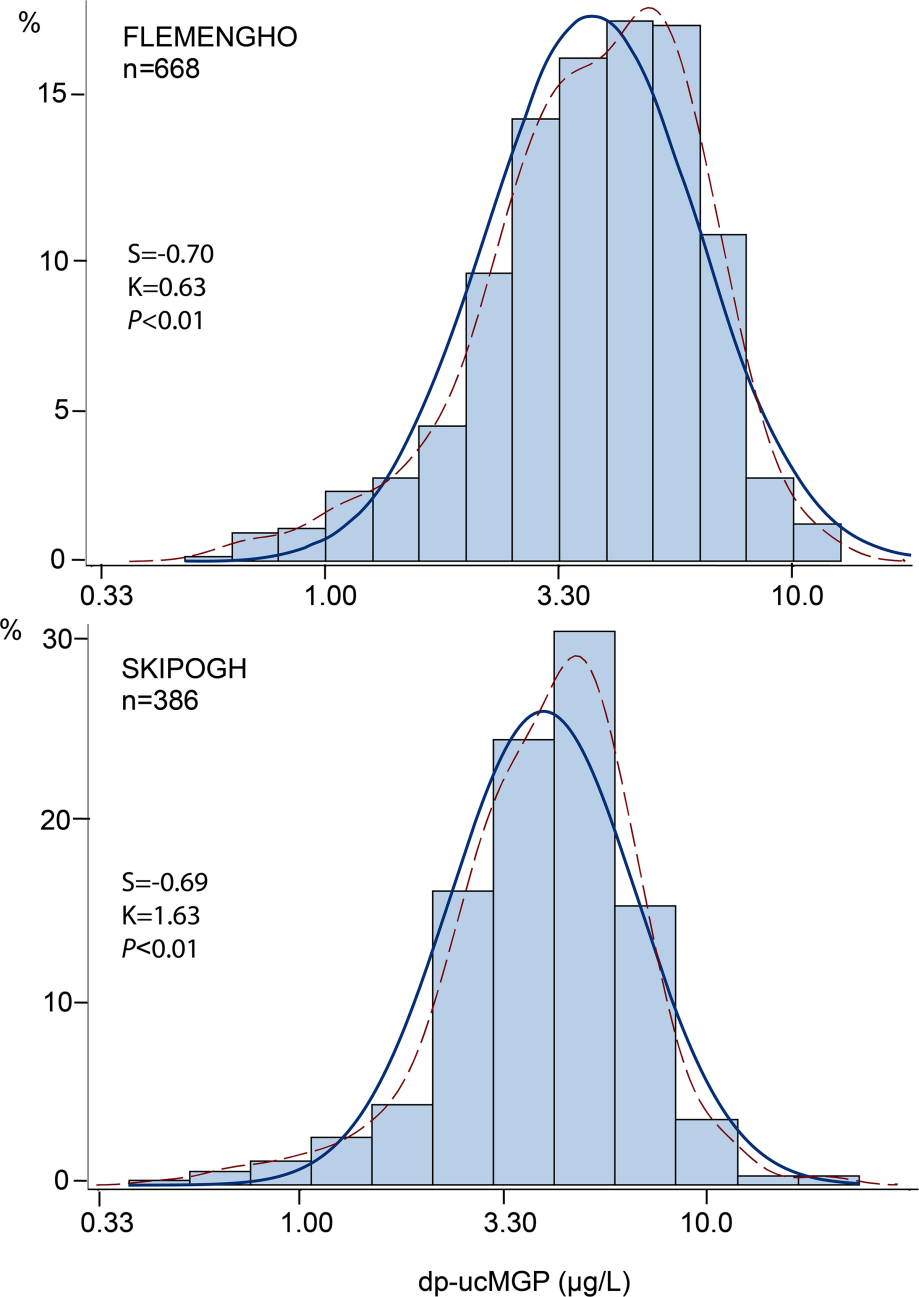
Frequency distributions of dp-ucMGP in Flemish and Swiss. S and K are the coefficients of skewness and kurtosis. The *P* value is for departure of the actually observed logarithmically transformed distribution (kernel distribution; dotted line) from normality (full line). The geometric mean concentration was 3.94 μg/L in Flemish and 4.20 μg/L in Swiss (*P* = 0.056).

The geometric mean concentration of dp-ucMGP was 3.94 μg/L (interquartile range, 2.89–5.76 μg/L; 5-95th percentile interval, 1.50–8.08 μg/L) in Flemish and 4.20 μg/L (interquartile range, 3.09–5.88 μg/L; 5-95th percentile interval, 1.65–8.74 μg/L) in Swiss (Fig 1). Table 1 lists the characteristics of participants by the cohort-specific median of the dp-ucMGP distribution. Age, body mass index, systolic and diastolic blood pressure, heart rate, the prevalence of hypertension and use of antihypertensive drugs, total cholesterol, plasma glucose, and serum creatinine all increased (*P*≤0.023) with higher dp-ucMGP category, whereas the opposite was the case for smoking (*P*<0.001). The distribution of these cardiovascular risk factors across categories of dp-ucMGP was consistent in Flemish, Swiss and all participants combined (Table 1).

**Table 1:** Characteristics of participants.

Characteristic	FLEMENGHO		SKIPOGH		All	
dp-ucMGP Threshold (μg/L)	≤4.16	>4.16	≤4.51	>4.51	≤4.29	>4.29
Number (%) with characteristics	334	334	194	192	527	527
Women (%)	163 (48.8)	172 (51.5)	101 (52.1)	109 (56.8)	265 (50.3)	280 (53.1)
Current smoker (%)	88 (26.4)	49 (14.7)‡	67 (34.5)	40 (20.8)†	153 (29.0)	91 (17.3)‡
Drinking alcohol (%)	243 (72.8)	219 (65.6)*	135 (69.6)	124 (64.6)	375 (71.2)	346 (65.6)
Diabetes mellitus (%)	6 (1.8)	14 (4.2)	1 (0.52)	6 (3.1)	8 (1.5)	19 (3.6)*
Hypertension (%)	92 (27.5)	162 (48.5)‡	24 (12.4)	58 (30.2)‡	119 (22.6)	217 (41.2)‡
Treated hypertension (%)	51 (15.3)	99 (29.6)‡	10 (5.2)	29 (15.1)†	64 (12.1)	125 (23.7)‡
Mean (±SD) of characteristics						
Age (years)	44.6±14.1	53.5±15.1‡	43.8±15.6	56.8±15.1‡	44.3±14.7	54.7±15.1‡
Body mass index (kg/m^2^)	25.1±4.1	27.4±4.4‡	24.2±4.0	26.7±4.6‡	24.8±4.0	27.2±4.5‡
Systolic pressure (mm Hg)	124.4±14.0	131.1±16.1‡	114.6±16.1	122.5±15.5‡	121.0±15.6	127.8±16.4‡
Diastolic pressure (mm Hg)	78.4±9.1	81.1±9.4‡	73.1±8.4	77.2±8.9‡	76.5±9.3	79.6±9.4‡
Pulse pressure (mm Hg)	46.0±11.2	50.1±14.1‡	41.5±12.7	45.4±12.2‡	44.6±11.9	48.2±13.7‡
Heart rate (beats/min)	62.0±9.1	64.4±9.4‡	65.0±9.5	67.1±11.2*	63.2±9.4	65.3±10.1‡
Total cholesterol (mmol/L)	5.07±0.90	5.29±0.96‡	4.78±0.98	5.26±1.10‡	4.97±0.94	5.27±1.02‡
Plasma glucose (mmol/L)	4.82±0.64	4.95±0.74*	4.82±0.57	5.21±1.17‡	4.82±0.62	5.04±0.93‡
Serum creatinine (μmol/L)	80.8±13.4	84.7±15.4‡	73.9±11.7	78.6±16.4‡	78.2±13.1	82.4±16.1‡

Values are mean (SD) or number of subjects (%). Diabetes was a fasting plasma glucose ≥7.0 mmol/L or use of antidiabetic drugs. Hypertension was a blood pressure of ≥140 mm Hg systolic or ≥90 mm Hg diastolic or use of antihypertensive drugs. Pulse pressure is the difference between systolic and diastolic blood pressure. *P* values indicate the significance of the differences across median of the matrix Gla protein distribution

* *P*≤0.05

† *P*≤0.01

‡ *P*≤0.001.

Table 2 shows the sex- and age-standardized echocardiographic measurements by the cohort-specific median of the dp-ucMGP distributions. In Flemish and in all participants combined, the A peak velocity and the E/e’ ratio increased (*P*≤0.026) with higher dp-ucMGP category, whereas the opposite was the case (*P*≤0.012) for the e’ peak velocities and the ratios E/A and e’/a’. In Swiss, trends were similar, but significance was only reached for the decrease in e’ (*P* = 0.002) and the increase in E/e’ (*P* = 0.007) with higher dp-ucMGP category (Table 2).

**Table 2:** Sex- and age-standardized echocardiographic measurements by median of dp-ucMGP distribution.

Measurement	FLEMENGHO		SKIPOGH			All		
dp-ucMGP threshold (μg/L)	≤4.16	>4.16	≤4.51	>4.51		≤4.29	>4.29	
Left ventricular structure	334	334	194	192		527	527	
Internal diameter (cm)	5.01±0.40	5.05±0.41	4.52±0.49	4.57±0.51		4.84±0.49	4.87±0.50	
Interventricular septum (cm)	0.98±0.14	0.97±0.15	0.88±0.35	0.90±0.38		0.93±0.24	0.95±0.26	
Posterior wall (cm)	0.89±0.12	0.90±0.12	0.80±0.28	0.81±0.13		0.86±0.19	0.86±0.13	
Relative wall thickness	0.37±0.06	0.37±0.06	0.40±0.43	0.41±0.47		0.38±0.27	0.38±0.29	
Mass index (g/m^2^)	89.4±21.1	92.6±20.5*	67.9±18.4	69.1±18.3		81.5±22.5	84.1±22.7	
Ejection fraction (%)	68.3±7.1	68.7±7.4	63.3±4.9	64.8±6.0†		66.5±6.8	67.2±7.1	
Doppler data								
A peak (cm/s)	61.1±12.6	64.0±13.6†	58.6±13.6	63.8±15.4		60.1±13.2	64.2±14.1†	
E peak (cm/s)	75.6±14.3	75.5±13.1	73.2±14.9	71.2±15.6		75.2±14.6	73.4±14.0	
E/A ratio	1.34±0.41	1.27±0.37*	1.36±0.48	1.23±0.35		1.36±0.43	1.25±0.36†	
e' peak (cm/s)	12.1±2.7	11.6±2.6†	12.0±2.3	10.7±2.4†		12.1±2.6	11.2±2.6‡	
a' peak (cm/s)	9.77±1.87	9.95±1.86*	9.36±1.72	9.59±1.74		9.60±1.85	9.87±1.82	
e'/a' ratio	1.38±0.56	1.28±0.49†	1.45±0.49	1.25±0.45		1.41±0.54	1.26±0.47‡	
E/e' ratio	6.60±1.47	6.94±1.50†	6.30±1.45	7.16±1.94†		6.50±1.49	7.02±1.69‡	

Values are mean (SD). Ejection fraction was available in 631 Flemish, 379 Swiss and 1010 all participants combined. *S*ignificance of the differences across halves of the matrix Gla protein distribution

* *P*≤0.05

† *P*≤0.01

‡ *P*≤0.001.

Single-model tests demonstrated that the multivariable-adjusted regression lines relating the echocardiographic indexes to dp-ucMGP were all coincident in Flemish and Swiss. In Flemish, Swiss and all participants combined, each doubling of dp-ucMGP was associated with increases in E/e’ by 0.26 (*P*<0.001), 0.33 (*P* = 0.004) and 0.31 (*P* = 0.026), respectively (Table 3). Considering the e’ denominator of the E/e’ ratio, the association sizes were –0.21 cm/s (*P* = 0.049) in Flemish, –0.43 cm/s (*P* = 0.001) in Swiss and –0.31 cm/s (*P*<0.001) in all participants. The E numerator was not associated with dp-ucMGP in any cohort (*P*≥0.217). For each doubling of dp-ucMGP, the transmitral A peak increased by 1.26 cm/s (*P* = 0.034) in Flemish and by 1.28 cm/s (*P* = 0.014) in both cohorts combined with a similar trend in Swiss (1.07 cm/s; *P* = 0.279); the e'/a' ratio decreased by 0.034 (*P* = 0.002) in both cohorts combined with a similar trend in Flemish (–0.029; *P* = 0.146) and in Swiss (–0.044; *P* = 0.079). The E/A ratio (*P*≥0.211) and the a’ peak (*P*≥0.582) were not associated with dp-ucMGP in any cohort.

**Table 3:** Multivariable-adjusted association of left ventricular diastolic function with dp-ucMGP.

Doppler Indices	FLEMENGHO (n = 668)		SKIPOGH (n = 386)		ALL (n = 1054)	
	Estimate (95%CI)	*P* Value	Estimate (95%CI)	*P* Value	Estimate (95%CI)	*P* Value
A peak, cm/s	1.26 (0.10 to 2.42)	0.034	1.07 (–0.88 to 3.03)	0.279	1.28 (0.26 to 2.31)	0.014
E peak, cm/s	0.87 (–0.51 to 2.25)	0.217	–0.09 (–2.23 to 2.05)	0.933	0.62 (–0.55 to 1.80)	0.298
e’ peak, cm/s	–0.21 (–0.41 to –0.0003)	0.049	–0.43 (–0.68 to –0.18)	0.001	–0.31 (–0.47 to –0.15)	<0.001
e'/a' ratio	–0.029 (–0.068 to 0.010)	0.146	–0.044 (–0.092 to 0.005)	0.079	–0.034 (–0.065 to –0.004)	0.002
E/e’ ratio	0.26 (0.12 to 0.40)	<0.001	0.33 (0.10 to 0.55)	0.004	0.31 (0.18 to 0.43)	0.026

Values are regression coefficients (95% confidence interval). Estimates accounted for family cluster were adjusted sex, age, body mass index, mean arterial pressure, pulse pressure, heart rate, total cholesterol, plasma glucose, left ventricular mass index, alcohol intake and antihypertensive drug treatment. Estimates combining Flemish and Swiss were additionally adjusted for center.

**Fig 2:**
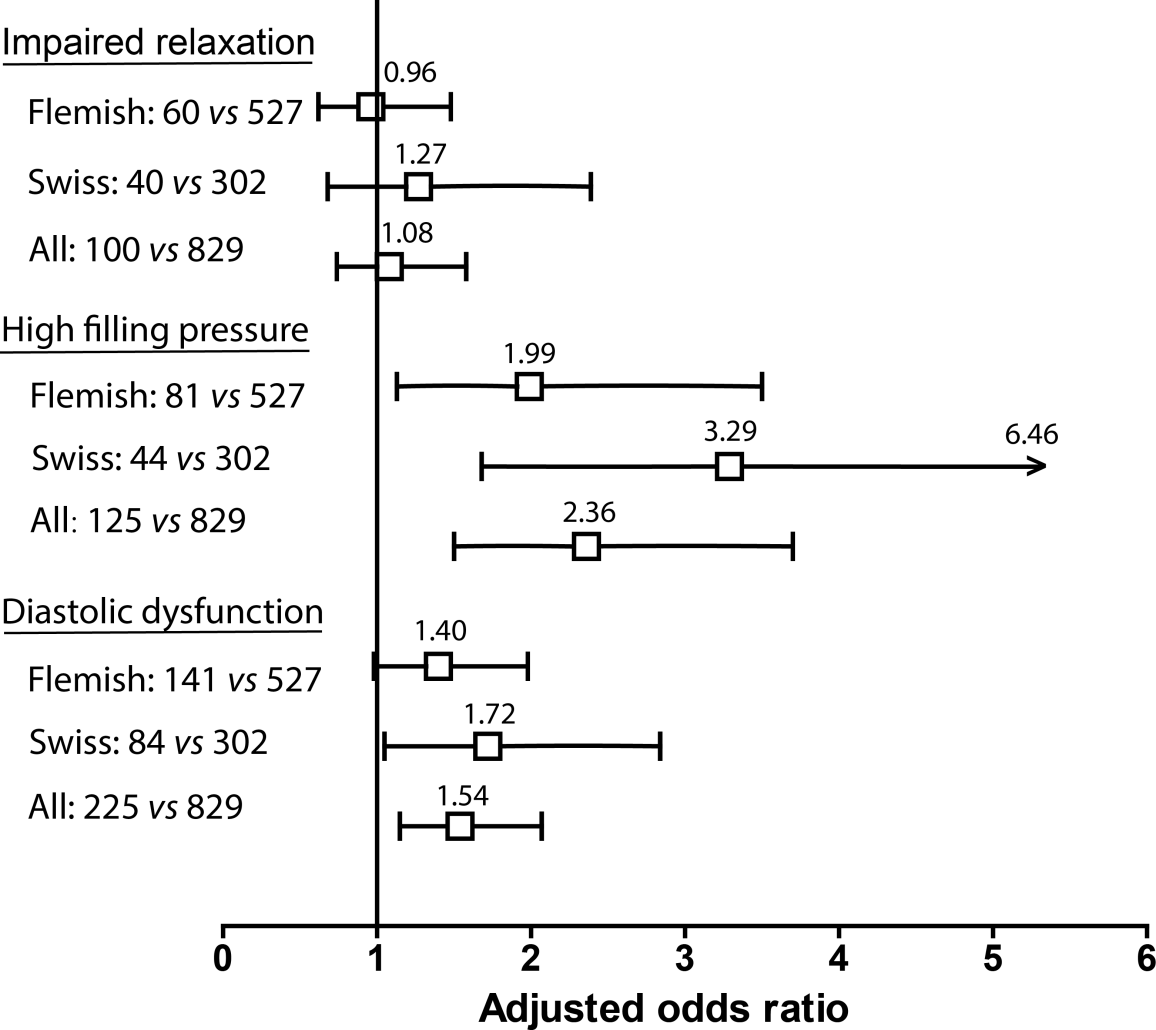
Odds ratios relating diastolic LV dysfunction to dp-ucMGP. For definition of the age-specific criteria of impaired relaxation and increased filling pressure, see Methods. The analyses accounted for cohort and family cluster and were adjusted for sex, age, body mass index, mean arterial pressure, pulse pressure, heart rate, total cholesterol, plasma glucose, left ventricular mass index, alcohol intake and antihypertensive drug treatment. Horizontal bars denote the 95% confidence interval. For each entry, the number of people with diastolic LV dysfunction *vs*. normal function are given.

In multivariable categorical analyses, impaired LV relaxation was not associated with dp-ucMGP in any cohort (0.96 ≤ odds ratio ≤ 1.27; *P*≥0.455; Fig 2). Increased LV filling pressure was associated with dp-ucMGP in all cohorts with odds ratios for a doubling of dp-ucMGP amounting to 1.99 (*P* = 0.017) in Flemish, 3.29 (*P*<0.001) in Swiss and 2.36 (*P*<0.001) in all participants (Fig 2). For impaired relaxation combined with increased LV filling pressure, the odds ratios were 1.40; *P* = 0.061) in Flemish, 1.72 (*P* = 0.032) in Swiss and 1.54 (*P* = 0.004) in all participants (Fig 2).

### Histological studies

All patients with heart disease and donors were white. Age at transplantation ranged from 20 to 66 years and from 59 to 63 years in patients with dilated or ischemic cardiomyopathy, respectively (Table 4). Age at the death among the donors ranged from 63 to 82 years. The causes of death in donors were hemorrhagic stroke in three and ischemic stroke in one. Age was the main criterion why donor hearts were not implanted. All patients with cardiomyopathy wore a cardiac resynchronization, defibrillator or left ventricular assist device before being transplanted (Table 4).

**Table 4:** Characteristics of patients with cardiomyopathy and donors.

Subjects	Age (years)	Sex	Device	LVEF (%)	LVDD (mm)	IVS (mm)	LVPW (mm)	ucMGP area (μm^2^/field)		pMGP Number	
								Mean	SEM	Mean	SEM
DCM	66	Man	CRT-D	30	68.0	9.9	4.1	84.00	25.32	37.33	3.28
DCM	65	Man	ICD	15	58.1	8.3	10.2	115.00	40.09	32.00	1.00
DCM	61	Man	CRT-D	22	54.7	13.6	10.7	91.50	24.78	36.67	1.76
DCM	20	Man	Heartmate II	15	70.2	8.4	7.9	129.75	34.83	42.00	2.08
ICM	63	Man	CRT-D	10	78.1	7.5	11.5	36.25	6.76	16.67	0.88
ICM	61	Man	CRT-D	34	51.4	11.2	11.4	24.75	12.64	30.67	2.73
ICM	59	Man	Heartware, CRT-D	15	76.5	4.4	7.9	252.25	73.42	15.00	2.00
ICM	61	Man	Heartmate II	10	66.5	7.4	8.0	901.75	161.59	40.33	2.31
Donor	71	Man	None	…	…	…	…	11.50	2.25	2.67	0.88
Donor	63	Man	None	…	…	…	…	5.50	2.36	8.67	1.33
Donor	82	Woman	None	…	…	…	…	16.50	4.57	10.00	1.73
Donor	75	Man	None	…	…	…	…	1.00	1.00	5.00	1.15

Abbreviations: DCM, dilated cardiomyopathy; ICM, ischaemic cardiomyopathy; CRT-D, cardiac resynchronisation therapy defibrillator; ICD, implantable cardioverter defibrillator; LVEF, left ventricular ejection fraction; LVDD, left ventricular end-diastolic diameter; IVS, Thickness of the interventricular septum at end-diastole; LVPW, thickness of the left ventricular posterior wall at end-diastole; ucMGP, uncarboxylated matrix Gla protein; pMGP, phosphorylated matrix Gla protein. Heartmate II (Thoratec Corporation, California, USA) is a left ventricular assist device implanted as bridge to heart transplantation. An ellipsis indicates data not available.

**Fig 3:**
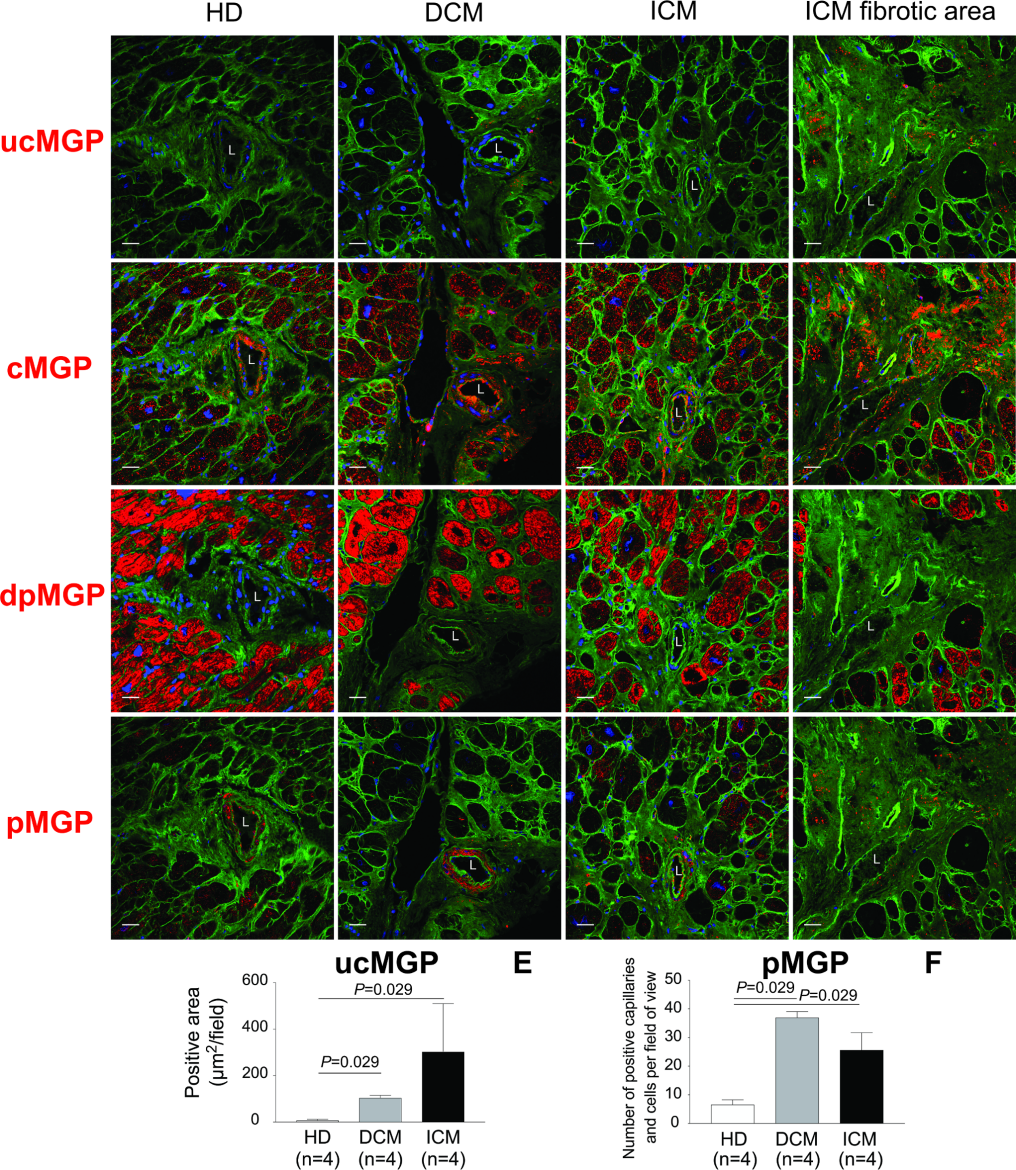
Immunofluorescent localization of MGP species in consecutive sections of the left ventricular myocardium. Rows are labelled by the MGP conformation (red) stained for: from top to bottom uncarboxylated MGP (ucMGP), carboxylated MGP (cMGP), unphosphorylated MGP (dpMGP) and phosphorylated MGP (pMGP). pMGP and cMGP include the secreted and active MGP conformation. Columns refer to exemplary tissue samples of male patients aged 61–63 years: from left to right unused donor heart (.h [HD]), dilated cardiomyopathy (.d [DCM] and ischemic cardiomyopathy representative section (.i [ICM]) and fibrotic area of the same ischemic heart (.f). WGA (green) and TO-PRO3 (blue) stain cell membranes and nuclei, respectively. The dilution of the conformation specific antibodies was 1:100 for ucMGP, dpMGP, pMGP and the inset in panel B.co and 1:250 for cMGP. The scale bar corresponds to 25 μm. The findings are described in a qualitative way in the results section. The quantitative analysis, comparing abundance of ucMGP and pMGP among HD, DCM and ICM appear in panels E and F, respectively. n refers to the number of tissue samples included in the quantitative analysis. Labels: asterisks indicate cardiomyocytes; arrows point to interstitial cells with perinuclear MGP deposition; L lumen of muscularized microvessels.

**Fig 4:**
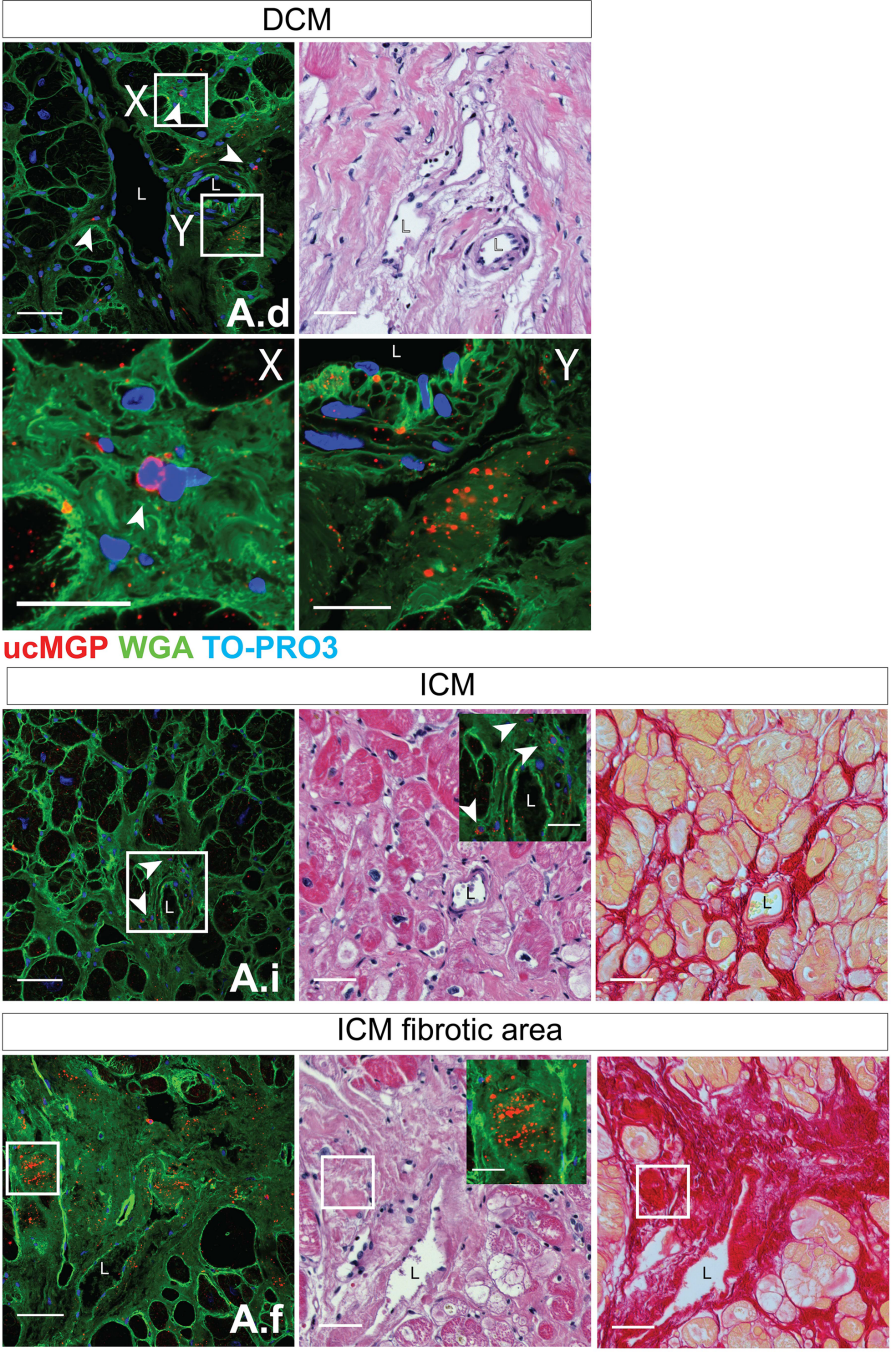
Fluorescence, H&E, Sirius Red staining and magnified ucMGP images of panels A.d (dilated cardiomyopathy [DCM]), A.i (ischemic cardiomyopathy [ICM]) and A.f (ICM fibrotic area) taken from Fig 3. The scale bar in A.d, A.i, A.f, H&E and Sirius Red images corresponds to 50 μm. Squares indicate areas that are magnified (scale bar 10 μm). X and Y label two magnified areas of DCM panel A.d. L indicates vessel lumen. White arrows point to ucMGP staining (red).

**Fig 5:**
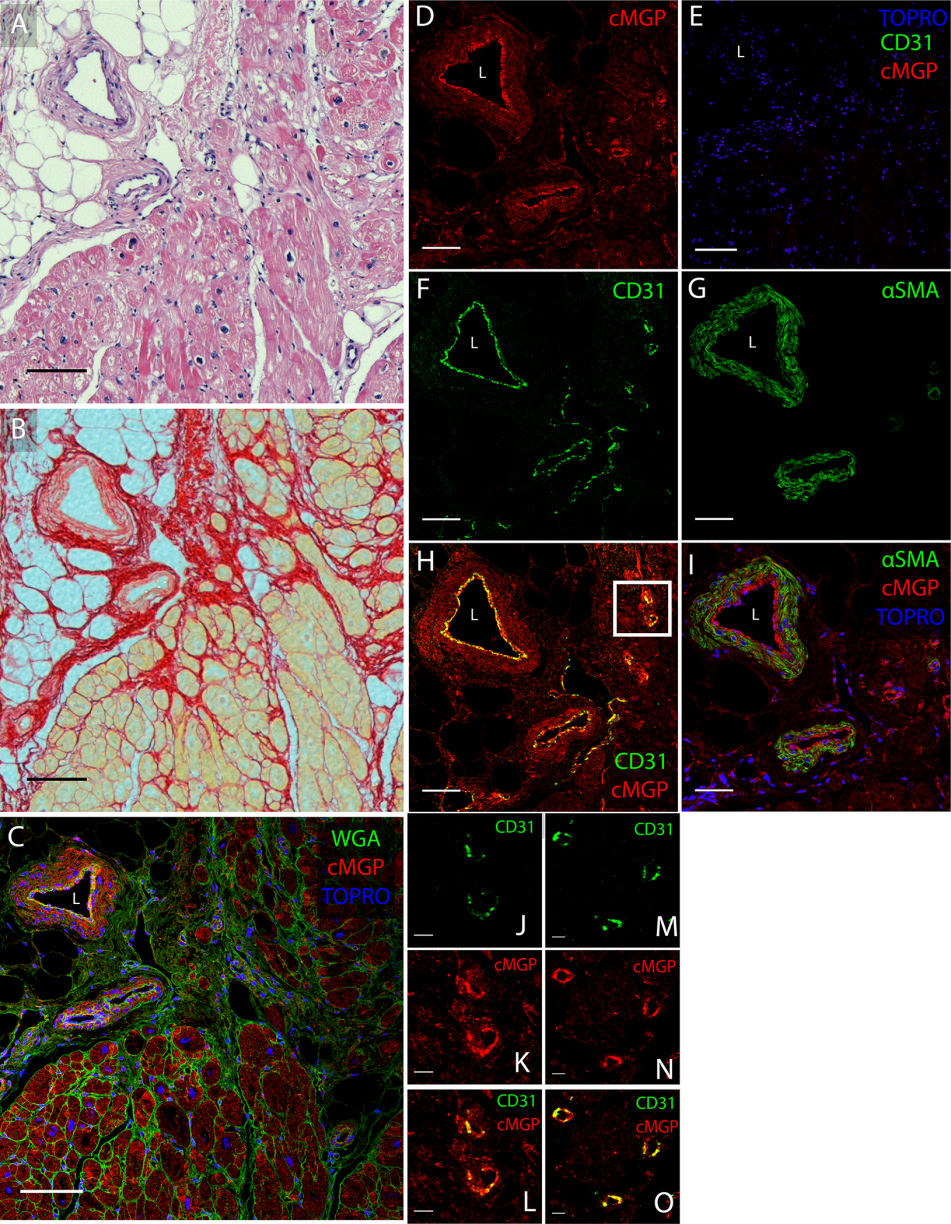
cMGP immunofluorescent localization in left ventricular myocardium in an older patient aged 61 years with dilated cardiomyopathy. H&E staining (A), Sirius Red staining (B), cMGP (red [D]), CD31 (green [F]), α-smooth muscle actin (αSMA, green [G]). Panels are multiple stains highlighting: (i) cMGP (red), cell membranes (WGA, green) and nuclei (TO-PRO3, blue) in panel C; (ii) cMGP (red) and endothelium (CD31, green) in panel H; (iii) cMGP (red), αSMA (green) and nuclei (TO-PRO3, blue) in panel I; and (iv) a negative control without cMGP and CD31 primary antibodies in panel E. Two sets of magnifications visualize the microvascular endothelium. Images J, K and L correspond to the square in panel H, while images M, N and O are taken from another area of panel H. cMGP is abundant in the small muscularized vessels (panels D, E, F, G, H and I), in microvascular endothelium (panels J, K, L, M, N and O) and in (panel C). The scale bar represents 100 μm in panels A, B and C; 50 μm in panels D, E, F, G, H and I; and 10 μm in panels J, K, L, M, N and O. L indicates vascular lumen.

**Fig 6:**
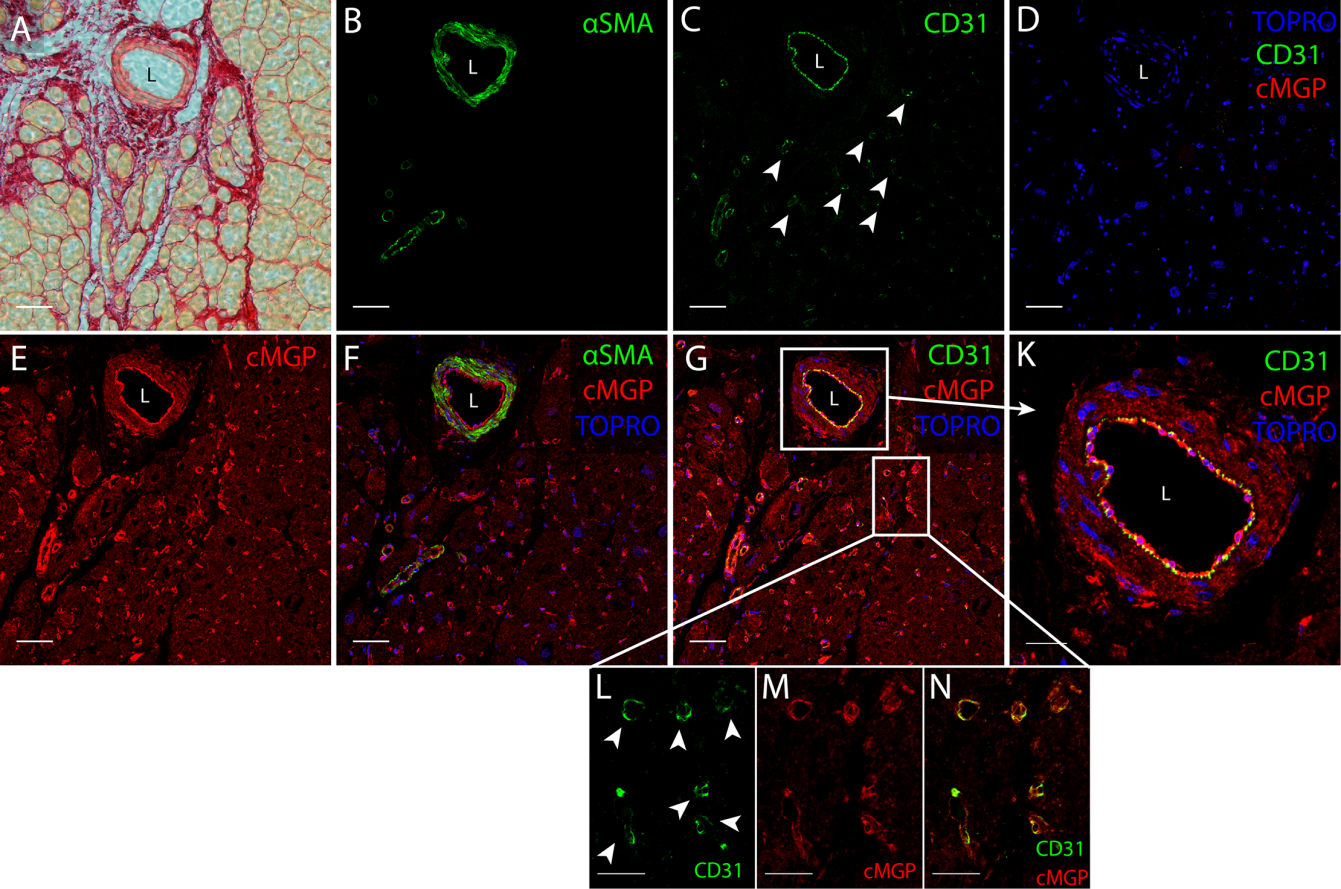
cMGP immunofluorescent localization in left ventricular myocardium in younger patient aged 20 years with dilated cardiomyopathy. Sirius Red staining (A), α-smooth muscle actin (αSMA, green [B]), CD31 (green [C]), cMGP (red [E]). Panels are triple stains highlighting: (i) cMGP (red), αSMA (green) and nuclei (TO-PRO3, blue) in panel F; (ii) cMGP (red), endothelium (CD31, green) and nuclei (TO-PRO3, blue) in panel G; and (iii) a negative control without cMGP and CD31 primary antibodies in panel D. The squares in panel G identify a muscularized vessel magnified in panel K and microvascular endothelium magnified in panels L, M and N. White arrows point to capillaries. cMGP deposition is comparable to the older patient in Fig 5. The scale bar represents 50 μm in panels A, B, C, D, E, F and G and 10 μm in panels K, L, M and N. L indicates vascular lumen.

**Fig 7:**
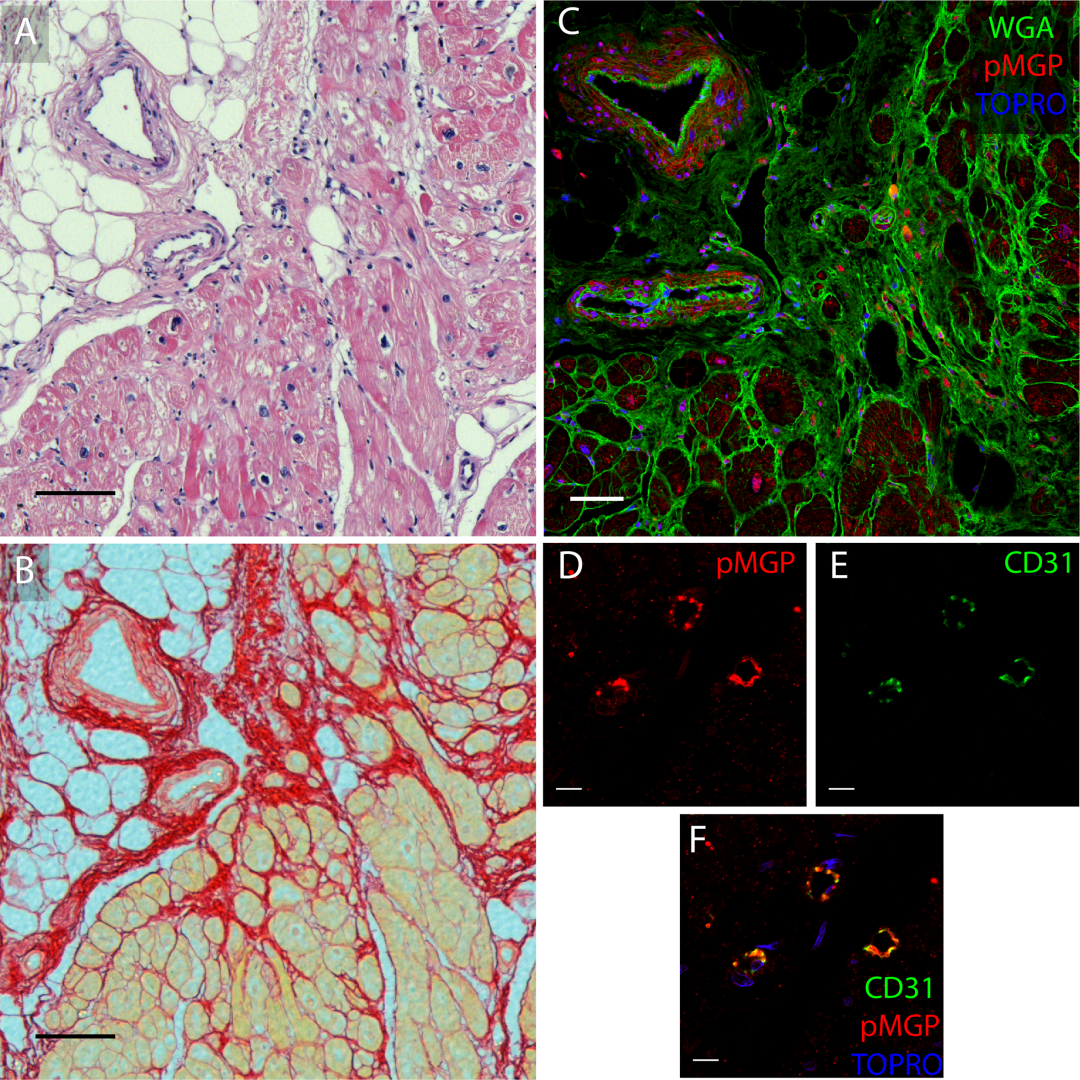
pMGP immunofluorescent localization in left ventricular myocardium in an older patient aged 61 years with dilated cardiomyopathy. H&E staining appears in panel A, Sirius Red staining in panel B, and staining for pMGP (red) and WGA (green) and TO-PRO3 (blue) in panel C. Magnifications of staining of capillaries appear in panel D for pMGP, in panel E for CD31 and in panel F for pMGP, CD31 and TO-PRO3. The scale bar represents 100 μm in panels A, B and C and 10 μm in panels D, E and F.

**Fig 8:**
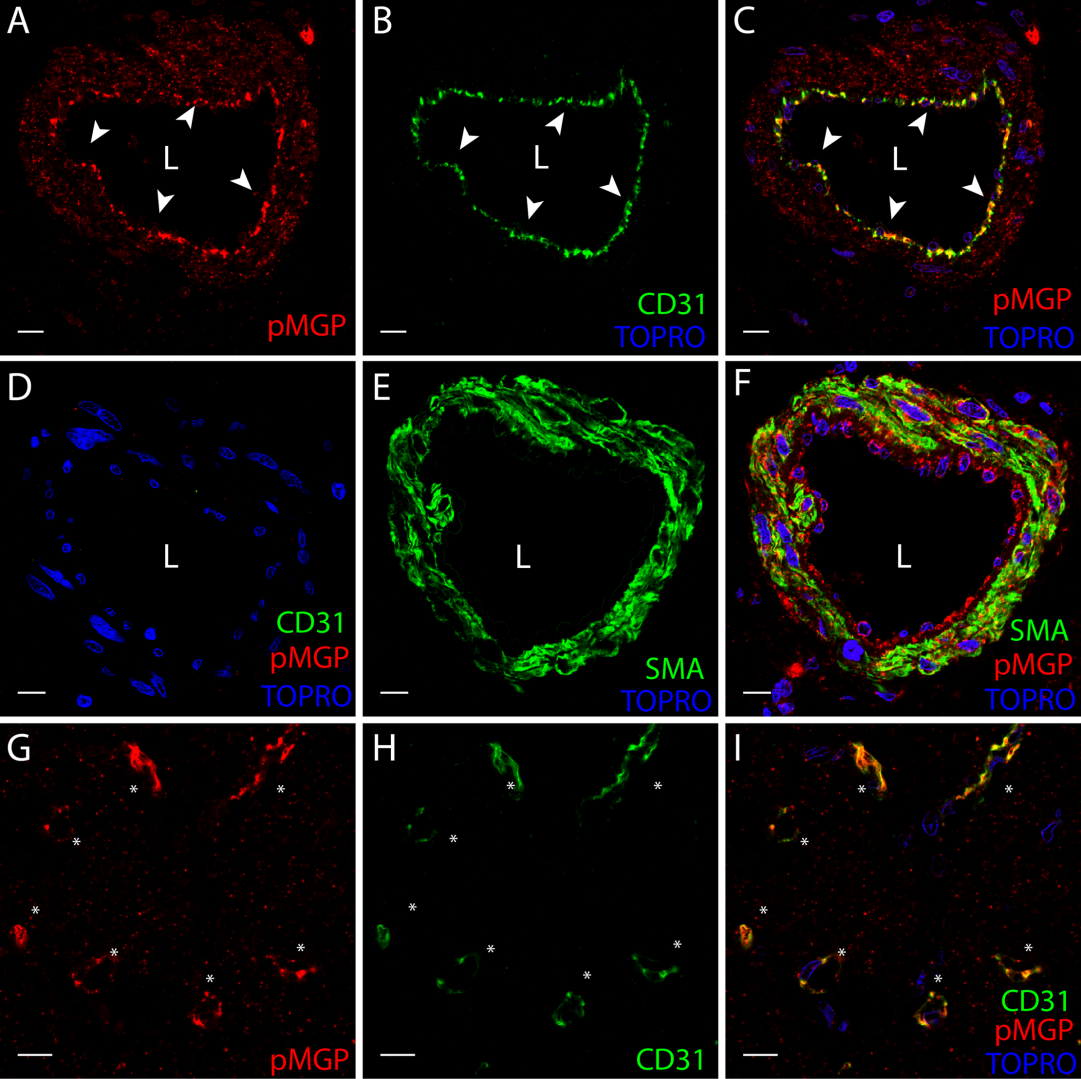
pMGP immunofluorescent localization in the left ventricular myocardium in younger patient aged 20 years with dilated cardiomyopathy. Staining for pMGP (red) appears in panels A and G, for CD31 (green) in panels B and H, for pMGP (red) and TO-PRO3 (blue) in panel C, and for α-smooth muscle actin (αSMA, green) in panel E. Triple stains highlight: (i) pMGP (red), αSMA (green) and nuclei (TO-PRO3, blue) in panel F; (ii) pMGP (red), endothelium (CD31, green) and nuclei (TO-PRO3, blue) in panel I; and (iii) a negative control without pMGP and CD31 primary antibodies in panel D. pMGP is abundant in the endothelium and vessel wall of muscularized vessels (panels A, B, C, D, E and F) and in capillaries (panels G, H and I). pMGP deposition is comparable to the old patient in Fig 7. White arrows point to endothelial layer. The scale bar represents 10 μm. L indicates the vessel lumen.

The active MGP moieties, cMGP (Fig 3B) and pMGP (Fig 3D), were predominantly distributed in the media and intima of muscular left ventricular microvessels in healthy and diseased hearts. Inactive ucMGP was abundant in fibrotic areas of diseased hearts, around the nuclei of interstitial cells and in the perivascular matrix (Fig 3A and Fig 4). ucMGP was more abundant in DCM and ICM than HD hearts (mean±SEM, 105.1±10.6 and 303.8±206.1 *vs*. 8.6±3.4 μm2/field; *P* = 0.029; Fig 3E). In ICM hearts, ucMGP was particularly present in fibrotic areas and, depending on the degree of fibrosis, showed large variability between patients (Table 4). ucMGP staining was absent in cardiomyocytes (Fig 3A). Furthermore, DCM and ICM myocardium showed more pMGP positive capillaries and interstitial cells than HD hearts (37.0±2.0 and 25.7±6.0 *vs*. 6.6±1.7; *P* = 0.029; Fig 3F and Table 4). Finally, staining for inactive dpMGP was almost absent in the vessel wall and in fibrotic areas, but was abundant in cardiomyocytes of all hearts and co-localized with active cMGP (Fig 3C). There were no differences in cMGP (Figs 5 and 6) and pMGP (Figs 7 and 8) staining between an older (61 years) and young (20 years) patient with DCM.

## Discussion

Paulus and Tschöpe proposed a novel paradigm that shifts emphasis in diastolic LV dysfunction from LV afterload to inflammation of the coronary microcirculation [2]. This paradigm [2] along with the premise that MGP is promoting the integrity of the microcirculation [11] and might be a local inhibitor of soft tissue calcification [12,13] provided the rationale for the hypothesis that echocardiographically assessed diastolic LV function might be inversely associated with circulating inactive dp-ucMGP. We confirmed our hypothesis in two population cohorts, while subsequent tissue staining studies revealed the localization of the active and inactive MGP moieties in the myocardium, thereby supporting a role of active MGP in maintaining a healthy myocardium.

**Fig 9:**
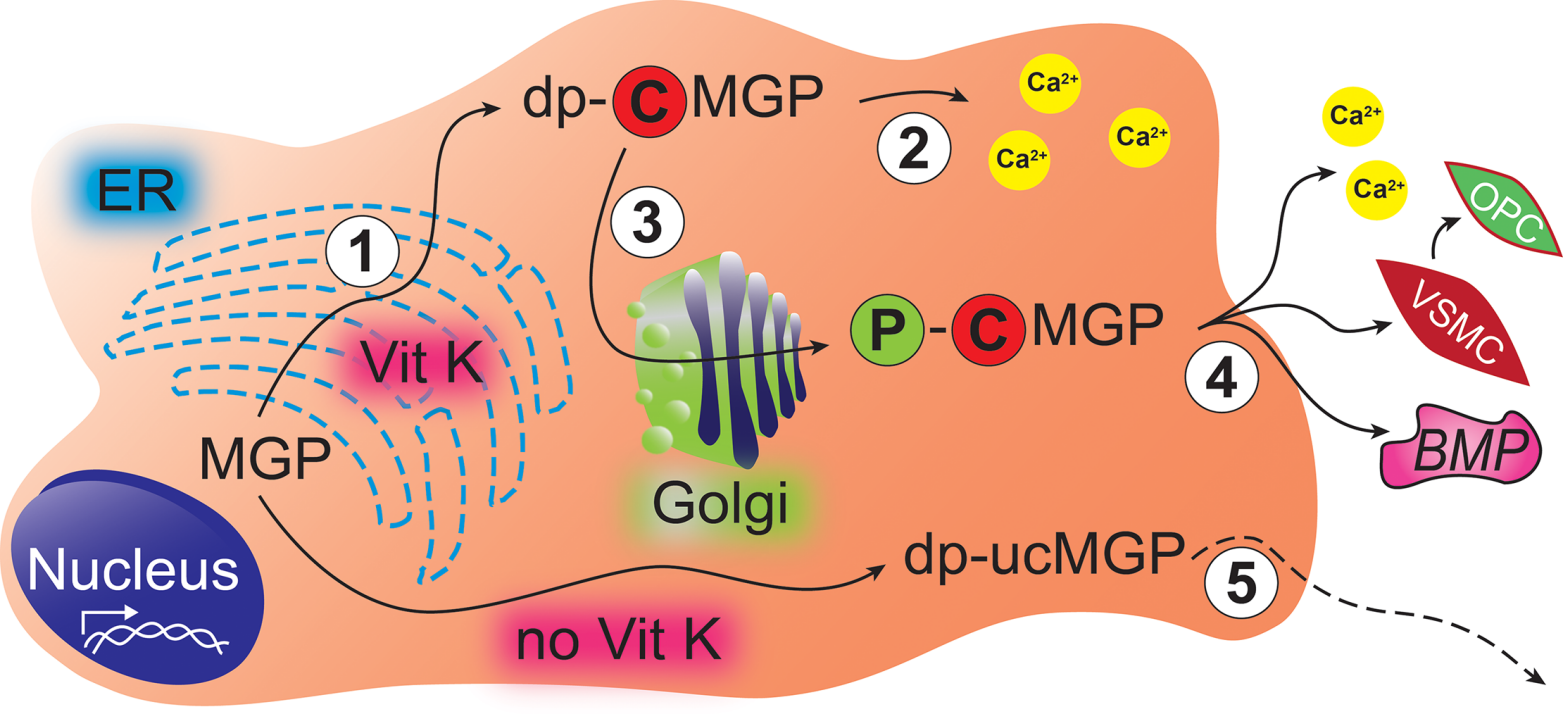
Hypothetical pathways aligning with the histological findings. Cardiomyocytes, interstitial, endothelial and vascular smooth muscle cells of the human heart express MGP. Step 1: After translation in the endoplasmatic reticulum (ER), vitamin K activates MGP by stimulating γ-carboxylation. Step 2: Carboxylated desphospho-MGP (dp-cMGP) can sequesters intracellular calcium, thereby providing protection against injury caused by calcium deposition. Step 3: A Golgi-associated casein kinase phosphorylates the serine residues of dp-cMGP to p-cMGP, thereby facilitating secretion. Step 4: p-cMGP is secreted into the extracellular matrix or the circulation to inhibit soft tissue calcification, vascular smooth muscle cell (VSMC) trans-differentiation into osteochondrogenic progenitor cells (OPC) and signaling via the BMP (bone morphogenetic protein) pathway. Step 5: Inactive desphospho-uncarboxylated MGP (dp-ucMGP), a biomarker reflecting poor vitamin K status, escapes from cells into the blood stream, but does not inhibit calcification.

In diseased compared with normal hearts, uncarboxylated MGP was more prevalent in the perivascular matrix and interstitium and phosphorylated MGP in and around capillaries and interstitial cells. Cardiomyocytes showed no staining for uncarboxylated MGP. Unphosphorylated MGP was almost absent in vessel walls and fibrotic areas, but was present in cardiomyocytes of all hearts and co-localized with carboxylated MGP. The ischemia-induced fibrotic remodeling in ICM patients seemed to be accompanied by an even higher deposition of ucMGP (Fig 3A and 3F). Fig 9 illustrates the hypothetical sequence of events possibly explaining our histological observations. Once secreted into the extracellular matrix, carboxylated and phosphorylated MGP protects against calcium deposition [22,23] and inhibits trans-differentiation of vascular smooth muscle cells [24] and signaling via the bone morphogenetic protein (BMP) pathway [25–27]. Taken together with the literature [22–27], our current findings support the idea that activated MGP is an ubiquitous locally acting agent protecting the microcirculation and the perivascular matrix.

Several mechanisms might explain how activated MGP might promote preservation of diastolic LV function. First, MGP binds to calcium ions as well as to hydroxyapatite crystals and may thereby inhibit crystal growth [22]. Depending on the amino-acid sequence, varying phosphorylated and γ-carboxylated sequences of human MGP inhibited either nucleation or growth, or both, of hydroxyapatite and calcium oxalate monohydrate crystals [23]. Relevant to the current study, is the strong well-documented protein-protein interaction between MGP and BMP, including BMP-2 [25,26] and BMP-4 [27], whereby bound MGP reduces BMP signaling. In the heart, BMP pathways play a pivotal role in the embryogenesis of the LV chamber [28], the differentiation of cardiac progenitor cells into functional cardiomyocytes [29], maintenance of the balance between LV growth and apoptosis [30], initiating fibrosis [30], and Ca2+ channel remodeling [31]. In line with the paradigm that the microcirculation is involved in the pathogenesis of diastolic LV function [2], BMP signaling also promotes endothelial apoptosis [32], increases endothelial permeability [32], and is involved in the expression of vascular endothelial growth factor and angiogenesis [27].

Circulating dp-ucMGP is a biomarker reflecting vitamin K deficiency [33]. Indeed, among 60 middle-aged healthy volunteers randomized in a placebo-controlled double-blind trial, plasma dp-ucMGP dropped dose-dependently by 31% and 46% in response to daily supplementation for 12 weeks with 180 μg and 360 μg of menaquinone-7 (vitamin K2) [33]. Selectively re-introducing MGP expression in the liver of MGP knockout mice, produced circulating MGP levels 6- to 10-fold higher than in wild type animals [34]. The MGP originating from the transgene conserved its biological activity in vitro, but did not inhibit arterial calcification [34], confirming that MGP is a locally acting paracrine protein. We did not measure circulating levels of vitamin K, which is rarely done in clinical practice, because of the complexity of the assay and the lack of a high-throughput method [35] and because plasma levels only reflect dietary intake (vitamin K1; phylloquinone) and production by the gut microflora (vitamin K2; menaquinones) without giving any indication of functionality, i.e. the amount of MGP undergoing carboxylation [33].

The clinical implications of our current findings rest on the prognostic significance of the E/e’ ratio [36–39]. Among 816 hypertensive patients with echocardiographic data randomized in the Anglo-Scandinavian Cardiac Outcomes Trial (ASCOT), 56 cardiac events occurred over 4.2 years. The E/e' ratio was the strongest predictor of a first cardiac event. Following adjustment for covariables, a one unit rise in the E/e' ratio was associated with 17% increment in risk of a cardiac event (95% confidence interval, 5% to 29%; *P* = 0.003) [38]. Similarly, among 406 patients with type-2 diabetes mellitus, the E/e’ ratio was an independent predictor of cardiovascular events occurring over 5.6 years, including 12 myocardial infarctions and 7 strokes. In analyses adjusted for sex and age, a one unit rise in the E/e' ratio was associated with 8% increment in risk (1% to 16%; *P* = 0.021), whereas the association with global LV strain was not significant (*P* = 0.913) [39]. Along similar lines, e’ predicted mortality in a study including 174 hypertensive patients and 78 age-matched controls [40]. Our current findings focusing on diastolic LV function, are in line with our previous report showing that high dp-ucMGP predicted adverse health outcomes, including total and cardiovascular mortality in 2318 FLEMENGHO participants followed-up for a median of 14.1 years [9].

Strong points of our current study are the consistency of the association of the E/e’ ratio with dp-ucMGP in two population cohorts and the histological evidence supporting the epidemiological observations. However, our current study must also be interpreted within the context of its potential limitations. First, our population studies had a cross-sectional design, which precludes direct causal inference. Second, we did not measure circulating dp-ucMGP in the patients enrolled in the histological studies and we cannot conclude to what extent the patients with end-stage DCM or ICM are representative for early-stage diastolic LV dysfunction as observed in the general population. Third, the sample size of histological studies was relatively small, but of the same order of magnitude as in other studies of the same nature [41,42]. Fourth, the location of MGP species was not confirmed by Western blots, because the antibodies available to us had never before been validated for use in such studies. Finally, for the histological studies, we used conformation-specific MGP antibodies, but we did not apply measurement of protein expression or mRNA.

## Conclusions

In the general population, E/e' increased with higher dp-ucMGP, a marker of vitamin K deficiency. The combined epidemiological and histological findings suggest that poor vitamin K status, as exemplified by higher plasma dp-ucMGP, represents a risk factor for diastolic LV dysfunction. Whether our current findings open new avenues to the prevention or treatment of diastolic LV dysfunction or its progression to HF remains to be established in randomized clinical trials of vitamin-K supplementation.

## References

[1] Bui AL, Horwich TB, Fonarow GC. Epidemiology and risk profile of heart failure. Nat Rev Cardiol. 2011; 8: 30–41. doi: 10.1038/nrcardio.2010.165 21060326 10.1038/nrcardio.2010.165 PMC3033496

[2] Paulus WJ, Tschöpe C. A novel paradigm for heart failure with preserved ejection fraction. J Am Coll Cardiol. 2013; 62: 263–71. doi: 10.1016/j.jacc.2013.02.092 23684677 10.1016/j.jacc.2013.02.092

[3] Owan TE, Hodge DO, Herges RM, Jacobsen SJ, Roger VL, Redfield MM. Trends in prevalence and outcome of heart failure with preserved ejection fraction. N Engl J Med. 2006; 355: 251–9. doi: 10.1056/NEJMoa052256 16855265 10.1056/NEJMoa052256

[4] Kuznetsova T, Herbots L, López B, Jin Y, Richart T, Thijs L, et al Prevalence of left ventricular diastolic dysfunction in a general population. Circ Heart Fail. 2009; 2: 105–12. doi: 10.1161/CIRCHEARTFAILURE.108.822627 19808325 10.1161/CIRCHEARTFAILURE.108.822627

[5] Kloch-Badelek M, Kuznetsova T, Sakiewicz W, Tikhonoff V, Ryabikov A, González A, et al Prevalence of diastolic left ventricular dysfunction in European populations based on cross-validated diagnostic thresholds. Cardiovascular Ultrasound. 2012; 10: 10 doi: 10.1186/1476-7120-10-10 22429658 10.1186/1476-7120-10-10 PMC3351014

[6] Kuznetsova T, Thijs L, Knez J, Cauwenberghs N, Petit T, Gu YM, et al Longitudinal changes in left ventricular diastolic dysfunction in a general population. Circ Cardiovasc Imaging. 2015; 8: e002882 doi: 10.1161/CIRCIMAGING.114.002882 25873723 10.1161/CIRCIMAGING.114.002882

[7] Schurgers LJ, Cranenburg ECM, Vermeer C, VitaK and Cardiovascular Research Institute (CARIM) MUMTN. Matrix gla-protein: the calcification inhibitor in need of vitamin K. Thromb Haemost. 2008; 100: 593–603. 18841280

[8] Pivin E, Ponte B, Pruijm M, Ackermann D, Guessous I, Ehret G, et al Inactive matrix gla-protein is associated with arterial stiffness in an adult population-based study. Hypertension. 2015; 66: 85–92. doi: 10.1161/HYPERTENSIONAHA.115.05177 25987667 10.1161/HYPERTENSIONAHA.115.05177

[9] Liu YP, Gu YM, Thijs L, Knapen MHJ, Salvi E, Citterio L, et al Inactive matrix gla protein is causally related to adverse health outcomes: a Mendelian randomization study in a Flemish population. Hypertension. 2015; 65: 463–70. doi: 10.1161/HYPERTENSIONAHA.114.04494 25421980 10.1161/HYPERTENSIONAHA.114.04494

[10] Knapen MHJ, Braam LAJLM, Drummen NEA, Bekers O, Hoeks APG, Theuwissen E, et al Low-dose menaquinone-7 supplementation improves vascular properties in healthy postmenopausal women. J Thromb Haemost. 2015; 113: 1135–44. 10.1160/TH14-08-0675 25694037

[11] Wei FF, Drummen NEA, Schutte AE, Thijs L, Jacobs L, Petit T, et al Vitamin K dependent protection of renal function in multi-ethnic population studies. EBioMed. 2016; 4: 162–9. 10.1016/j.ebiom.2016.01.011 PMC4776057 26981580

[12] Wei FF, Drummen NEA, Thijs L, Jacobs L, Herfs M, van't Hoofd C, et al Vitamin-K-dependent protection of the renal microvasculature: histopathological studies in normal and diseased kidneys. Pulse. 2016; 4: 85–91. doi: 10.1159/000448008 27752480 10.1159/000448008 PMC5052692

[13] Wei FF, Thijs L, Zhang ZY, Jacobs L, Yang WY, Salvi E, et al The risk of nephrolithiasis is causally related to inactive matrix Gla protein, a marker of vitamin K status: a Mendelian randomization study in a Flemish population. Nephrol Dial Transplant. 2017; doi: 10.1093/ndt/gfx014 10.1093/ndt/gfx014 28340119

[14] Bers DM. Calcium fluxes involved in control of cardiac myocyte contraction. Circ Res. 2000; 87: 275–81. 10948060 10.1161/01.res.87.4.275

[15] Zhang ZY, Staessen JA, Thijs L, Gu Y, Liu Y, Jacobs L, et al Left ventricular diastolic function in relation to the urinary proteome: a proof-of-concept study in a general population. Int J Cardiol. 2014; 176: 158–65. doi: 10.1016/j.ijcard.2014.07.014 25065337 10.1016/j.ijcard.2014.07.014 PMC4155932

[16] Fraser JD, Price PA. Lung, heart, and kidney express high levels of mRNA for the vitamin K-dependent matrix Gla protein. Implications for the possible functions of matrix Gla protein and for the tissue distribution of the γ-carboxylase. J Biol Chem. 1988; 263: 11033–6. 3042764

[17] World Medical Association. World Medical Association Declaration of Helsinki. Ethical principles for medical research involving human subjects. JAMA. 2013; 310: 2191–4. doi: 10.1001/jama.2013.281053 24141714 10.1001/jama.2013.281053

[18] Mor-Avi V, Lang RM, Badano LP, Belohlavek M, Cardim NM, Derumeaux G, et al Current and evolving echocardiographic techniques for the quantitative evaluation of cardiac mechanics: ASE/EAE concensus statement on methodology and indications. Endorsed by Japanese Society of Echocardardiography. J Am Soc Echocardiogr. 2011; 24: 277–313. doi: 10.1016/j.echo.2011.01.015 21338865 10.1016/j.echo.2011.01.015

[19] Gottdiener JS, Bednarz J, Devereux R, Gardin J, Klein A, Manning WJ, et al American Society of Echocardiography recommendations for use of echocardiography in clinical trials. A report from the American Society of Echocardiography's Guidelines and Standard Committee and the Task Force on Echocardiography in Clinical Trials. J Am Soc Echocardiogr. 2004; 17: 1086–119. 10.1016/j.echo.2004.07.013 15452478

[20] Pruijm MT, Wuerzner G, Glatz N, Alwan H, Ponte B, Ackermann D, et al A new technique for simultaneous validation of two manual nonmercury auscultatory sphygmomanometers (A&D UM-101 and Accoson Greenlight 300) based on the international protocol. Blood Press Monit. 2010; 15: 322–5. doi: 10.1097/MBP.0b013e32833f56a8 20827175 10.1097/MBP.0b013e32833f56a8

[21] Cranenburg ECM, Koos R, Schurgers LJ, Magdeleyns EJ, Schoonbrood TH, Landewe RB, et al Characterisation and potential diagnostic value of circulating matrix Gla protein (MGP) species. Thromb Haemost. 2010; 104: 811–22. doi: 10.1160/TH09-11-0786 20694284 10.1160/TH09-11-0786

[22] Schurgers LJ, Uitto J, Reutelingsperger CP. Vitamin K-dependent carboxylation of matrix GLA-protein: a crucial switch to control ectopic mineralization. Trends Mol Med. 2013; 19: 217–26. doi: 10.1016/j.molmed.2012.12.008 23375872 10.1016/j.molmed.2012.12.008

[23] Goiko M, Dierolf J, Gleberzon JS, Liao Y, Grohe B, Goldberg HA, et al Peptides of matrix gla protein inhibit nucleation and growth of hydroxyapatite and calcium oxalate monohydrate crystals. PLoS One. 2013; 8: e80344 doi: 10.1371/journal.pone.0080344 24265810 10.1371/journal.pone.0080344 PMC3827180

[24] Speer MY, Yang HY, Brabb T, Leaf E, Look A, Lin WL, et al Smooth muscle cells give rise to osteochondrogenic precursors and chondrocytes in calcifying arteries. Circ Res. 2009; 104: 733–41. doi: 10.1161/CIRCRESAHA.108.183053 19197075 10.1161/CIRCRESAHA.108.183053 PMC2716055

[25] Wallin R, Cain D, Hutson SM, Sane DC, Loeser R. Modulation of the binding of matrix Gla protein (MGP) to bone morphogenetic protein-2 (BMP-2). Thromb Haemost. 2000; 84: 1039–44. 11154111

[26] Zebboudj AF, Imura M, Boström K. Matrix GLA protein, a regulatory protein for bone morphogenetic protein-2. J Biol Chem. 2002; 277: 4388–94. doi: 10.1074/jbc.M109683200 11741887 10.1074/jbc.M109683200

[27] Yao Y, Zebboudj AF, Shao E, Perez M, Boström K. Regulation of bone morphogenetic protein-4 by matrix GLA protein in vascular endothelial cells involves activin-like kinase receptor 1. J Biol Chem. 2006; 281: 33921–30. doi: 10.1074/jbc.M604239200 16950789 10.1074/jbc.M604239200

[28] Han P, Bloomekatz J, Ren J, Zhang R, Grinstein JD, Zhao L, et al Coordinating cardiomyocyte interactions to direct ventricular chamber morphogenesis. Nature. 2016; 534: 700–4. doi: 10.1038/nature18310 27357797 10.1038/nature18310 PMC5330678

[29] Cagavi E, Bartulos O, Suh CY, Sun B, Yue Z, Jiang Z, et al Functional cardiomyocytes derived from Isl1 cardiac progenitors via BMP4 stimulation. PLoS One. 2014; 9: e110752 doi: 10.1371/journal.pone.0110752 25522363 10.1371/journal.pone.0110752 PMC4270687

[30] Sun B, Huo R, Sheng Y, Li Y, Xie X, Chen C, et al Bone morphogenetic protein-4 mediates cardiac hypertrophy, apoptosis, and fibrosis in experimentally pathological cardiac hypertrophy. Hypertension. 2013; 61: 352–60. doi: 10.1161/HYPERTENSIONAHA.111.00562 23248151 10.1161/HYPERTENSIONAHA.111.00562

[31] Hu CW, Li Q, Zhang Y, Li YH, Jiang HC, Liu MY, et al Bone morphogenetic protein-4 induces upregulation of Cav3.1 Ca^2+^ channels in HL-1 atrial myocytes. Pflügers Arch—Eur J Physiol. 2014; 466: 2049–57. 24510064 10.1007/s00424-014-1459-5

[32] Wiley DM, Jin SW. Bone morphogenetic protein functions as a context-dependent angiogenic cue in vertebrates. Semin Cell Dev Biol. 2011; 22: 1012–8. doi: 10.1016/j.semcdb.2011.10.005 22008724 10.1016/j.semcdb.2011.10.005 PMC3548572

[33] Dalmeijer GW, van der Schouw YT, Magdeleyns E, Ahmed N, Vermeer C, Beulens JW. The effect of menaquinone-7 supplementation on circulating species of matrix Gla protein. Atherosclerosis. 2012; 225: 397–402. doi: 10.1016/j.atherosclerosis.2012.09.019 23062766 10.1016/j.atherosclerosis.2012.09.019

[34] Murshed M, Schinke T, McKee MD, Karsenty G. Extracellular matrix mineralization is regulated locally; different roles of two gla-containing proteins. J Cell Biol. 2004; 165: 625–30. doi: 10.1083/jcb.200402046 15184399 10.1083/jcb.200402046 PMC2172384

[35] Riphagen IJ, van der Molen JC, van Faassen M, Navis G, de Borst MH, Muskiet FAJ, et al Measurement of plasma vitamin K1 (phylloquinone) and K2 (menaquinones-4 and -7) using HPLC-tandem mass spectrometry. Clin Chem Lab Med. 2016; 54: 1201–10. doi: 10.1515/cclm-2015-0864 26630696 10.1515/cclm-2015-0864

[36] Okura H, Takada Y, Kubo T, Iwata K, Mizoguchi S, Taguchi H et al Tissue Doppler-derived index of left ventricular filling pressure, E/ E', predicts survival of patients with non-valvular atrial fibrillation. Heart. 2006; 92: 1248–52. doi: 10.1136/hrt.2005.082594 16449507 10.1136/hrt.2005.082594 PMC1861171

[37] Hillis GS, Møller JE, Pelikka PA, Gersh BJ, Wright RS, Omnen SR, et al Noninvasive estimation of left ventricular filling pressure by E/e' is a powerful predictor of survival after myocardial infarction. J Am Coll Cardiol. 2004; 43: 360–7. doi: 10.1016/j.jacc.2003.07.044 15013115 10.1016/j.jacc.2003.07.044

[38] Sharp AS, Tapp RJ, Thom SA, Francis DP, Hughes AD, Stanton AV, et al Tissue Doppler E/E' ratio is a powerful predictor of primary cardiac events in a hypertensive population: an ASCOT substudy. Eur Heart J. 2010; 31: 747–52. doi: 10.1093/eurheartj/ehp498 19942604 10.1093/eurheartj/ehp498

[39] Blomstrand P, Engvall M, Festin K, Lindstrõm T, Länne T, Maret E, et al Left ventricular diastolic function, assessed by echocardiography and tissue Doppler imaging, is a strong predictor of cardiovascular events, superior to global left ventricular longitudinal strain, in patients with type 2 diabetes. European Heart Journal—Cardiovascular Imaging. 2015; 16: 1000–7. doi: 10.1093/ehjci/jev027 25750201 10.1093/ehjci/jev027

[40] Wang M, Yip GW, Wang AY, Zhang Y, Ho PY, Tse MK, et al Tissue Doppler imaging provides incremental prognostic value in patients with systemic hypertension and left ventricular hypertrophy. J Hypertens. 2005; 23: 183–91. 15643141 10.1097/00004872-200501000-00029

[41] Venardos N, Bennett D, Weyant MJ, Reece TB, Meng X, Fullerton DA. Matrix Gla protein regulates calcification of the aortic valve. J Surg Res. 2015; 199: 1–6. doi: 10.1016/j.jss.2015.04.076 25990696 10.1016/j.jss.2015.04.076 PMC4604002

[42] Schurgers LJ, Teunissen KJF, Knapen MHJ, Kwaijtaal M, van Diest R, Appels A, et al Novel conformation-specific antibodies against matrix γ-carboxyglutamic acid (Gla) protein: Undercarboxylated matrix Gla protein as marker for vascular calcification. Arterioscler Thromb Vasc Biol. 2005; 25: 1629–33. doi: 10.1161/01.ATV.0000173313.46222.43 15961706 10.1161/01.ATV.0000173313.46222.43

